# Effect of Pericytes on Cerebral Microvasculature at Different Time Points of Stroke

**DOI:** 10.1155/2021/5281182

**Published:** 2021-12-22

**Authors:** Zhong Di, Xin Wu, Wei Xie, Xianming Lin

**Affiliations:** ^1^Department of Acupuncture and Moxibustion, The Third Affiliated Hospital of Zhejiang Chinese Medical University, Hangzhou, China; ^2^Department of Acupuncture and Moxibustion, Zhejiang Chinese Medical University Affiliated Four Provincial Bordering Hospital, Quzhou, China; ^3^The Third School of Clinical Medicine, Zhejiang Chinese Medical University, Hangzhou, China; ^4^Department of Acupuncture and Moxibustion, Quzhou Hospital of Traditional Chinese Medicine, Quzhou, China

## Abstract

Pericyte, as an important component of the blood-brain barrier, has received increasing attention in the study of cerebrovascular diseases. However, the mechanism of pericytes after the occurrence of cerebral ischemia is controversial. On the one hand, the expression of pericytes increases after cerebral ischemia, constricting the blood vessels to restrict blood supply and aggravating the damage caused by ischemia; on the other hand, pericytes participate in capillary angiogenesis in the ischemic area, which facilitates the repair of the ischemic injury area. The multifunctionality of pericytes is an important reason for this phenomenon, but the different time points of observation for the outcome indicators in each study are also an important factor that leads to the controversy of pericytes. Based on the review of a large database of original studies, the authors' team summarized the effects of pericytes on cerebral microvasculature at different time points after stroke, searched the possible markers, and explored possible therapeutic.

## 1. Introduction

Pericyte is an important component of the blood-brain barrier and plays an important role in maintaining the structural stability and normal function of the BBB [1, 2]. Under the condition of ischemic and hypoxic, pericytes involve in cerebral blood flow regulation, BBB destruction, and angiogenesis and have a significant role in the development of stroke disease [3]. Pericytes are multifunctional cells that express different function in different stages of stroke disease development [4].

Pericytes express *α*-smooth muscle actin (*α*-SMA) that controls changes in vascular diameter, diastolic, and vasodilatory and regulates cerebral blood flow [5]. In normal condition, pericytes regulate changes in microbleeds precisely through dynamic contraction and relaxation [6]. In ischemic and hypoxic conditions, pericytes are very sensitive to extracellular stimulus signals, resulting in pericyte dysfunction which leads to excessive contraction and causing impaired red blood cell passage followed by impaired microcirculation. Yemisci et al. found that the middle cerebral artery successfully reopened after 2 hours of ischemia while pericytes still contracted, and pericyte contraction caused capillary constriction, impeded red blood cell flow, and aggravated the ischemic and hypoxic state of brain tissue [7]. Excessive and sustained pericyte constriction may be responsible for local microcirculatory disturbances and is an important pathological mechanism in the early stages of cerebral ischemia.

Pericytes are most likely involved in the neovascularization and revascularization after cerebral ischemia by regulating the formation and maturation of neovascularization [8]. At 28 days after focal ischemia, new vessels could be observed in the peri-ischemic region and scar tissue area, demonstrating that neovascularization occurs after ischemia [9]. After ischemia and hypoxia, with the activation of vascular endothelial cells at the edge of the ischemic area, pericytes are recruited around the neovascularization of the ischemic area to promote the maturation and stabilization of the vessels [10]. Pericytes regulate angiogenesis and involve multiple signaling pathways, including platelet-derived growth factor *β* (PDGF-*β*), platelet -derived growth factor receptor *β* (PDGFR-*β*) [11], vascular endothelial growth factor (VEGF), vascular endothelial growth factor receptor (VEGFR) [12], transforming growth factor-*β* (TGF-*β*), and transforming growth factor receptor-*β* (TGF*β*R-2).

The function of pericytes in stroke can be summarized in two major aspects: injury and repair. Previous scholars summarized the functional changes of pericytes at different stages after stroke [13], but there is no clear statement on the temporal boundaries of pericyte changes on cerebral microvascular effects at different stages after stroke. Because the functions of pericytes at different stages are different, the corresponding markers of pericytes are also various. In this paper, based on the original published studies so far, we observe the different effects of pericyte functional changes on cerebral microvasculature at different time points after the establishment of the middle cerebral artery ischemia model in each study to provide a basis for future studies.

## 2. Methods

### 2.1. Search Strategies

We searched PubMed, EMBASE, and China National Knowledge Infrastructure (CNKI) from issue to September 2021. The following search phrases were used: “pericyte (MeSH Terms) OR pericyte (Title/Abstract)” AND “cerebral ischemia OR hemorrhagic stroke OR ischemic stroke OR cerebral I/R.” In addition, the reference lists of possible articles were searched to find relevant studies. All studies were limited to animal experiments.

### 2.2. Eligibility Criteria

Studies that met the following criteria were included in this analysis: (1) animal studies that established cerebral ischemia or cerebral ischemia-reperfusion models, excluding case reports, cell studies, human studies, and reviews. (2) Included studies must contain a model group of cerebral ischemia without any intervention. (3) The model animals were normal animals without hypertension, diabetes, transgenic, and other basic diseases. (4) Outcome indicators mainly observed pericyte morphology and pericyte markers. No animal model of brain I/R injury or no predetermined outcome was studied, excluding other additional treatments.

### 2.3. Data Extraction

The following details were extracted from the relative studies by two independent authors: (1) name of first author and year of publication; (2) animal information, including species, sex, number, and weight; (3) method of brain I/R model establishment, including brain ischemia time and reperfusion duration; (4) time of outcome index collection; and (5) pericyte-related outcome index data. The results of the tests at different time points were recorded in detail, and the data of the final tests were collected.

### 2.4. Risk of Bias in Individual Studies

Risk of bias was assessed by two authors using a 10-item scale [14]. The methods were as follows: (A) peer-reviewed publications, (B) temperature control, (C) random assignment of treatments or controls, (D) model-blind induction, (E) blinded assessment of outcomes, (F) use of anesthetics without significant intrinsic cardioprotective effects, (G) appropriate animal models (elderly, diabetic, or hypertensive), (H) sample size calculation, (I) compliance with animal welfare regulations, and (J) potential conflict-of-interest statements. One point was awarded for each item. Two authors independently assessed the quality of the study.

### 2.5. Statistical Analysis

Endnote X9 saved the literature that met the inclusion criteria and created a database. Use Excel to make tables and record the extracted data including studies, years, animals, models, ischemic duration, and outcome indicators.

## 3. Results

### 3.1. Study Selection

**Figure 1:**
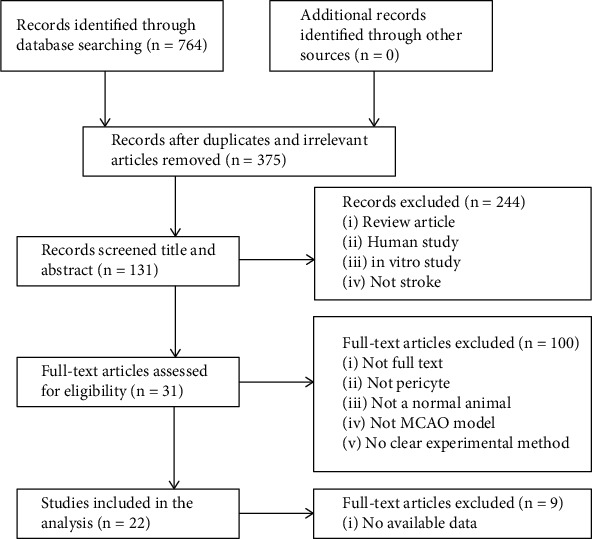
Summary of the process for identifying candidate studies.

A total of 764 articles were found by searching the database for relevant literature, of which 389 were duplicates and irrelevant. After screening titles and abstracts, 244 were excluded due to one of below reasons: (1) review articles, (2) human studies, (3) in vitro experiments, and (4) nonstroke. We studied the remaining 131 full-text articles and then eliminated 100 articles for at least one of the following reasons: (1) not full text, (2) not studying pericytes, (3) not normal animals, (4) not a model of middle cerebral artery obstruction, and (5) not telling the experimental methods clearly. Finally, 9 articles without available outcome indicators on pericytes were excluded, and 22 studies were selected at last [7, 15–35] (Figure 1).

### 3.2. Study Quality

The detailed results of methodological quality are listed in Table 1. The study quality scores ranged from 3 to 7, with a total score of 10. One study scored 3 [19]; 8 studies scored 4 [7, 16–18, 20, 26, 29, 34]; the quantities of studies scoring 5 [15, 25, 27, 28, 31, 35] and 6 [21–24, 30, 33] are both 6; one [32] study scored 7. All included studies were peer-reviewed publications, and all animals were randomized. No studies reported blinded modeling, blinded assessment of outcomes, or calculation of sample size. All of the studies were performed on healthy rats or mice. 17 studies described temperature control. 17 studies reported compliance with animal welfare regulations, and 6 studies declared no potential conflicts of interest. None of the studies used anesthetics with intrinsic cardioprotective properties.

### 3.3. Characteristics of Included Studies

A total of 22 papers were included in this study, 20 papers in English and 2 papers in Chinese [29, 34]. These 22 studies involved adult Sprague-Dawley (SD) rats [18, 23–26, 28, 30, 31], Wistar rats [22, 27, 29], and mouse [7, 15–17, 19–21, 32–35]. Adult rats weighed between 200 and 600 g, and mice weighed between 18 and 35 g. All studies used male animals. Three studies [16, 17, 34] used chloral hydrate to induce anesthesia; 5 studies [15, 19, 22, 32, 33] used halothane; 6 studies used isoflurane [20, 23–25, 27, 30]; the rest of the studies used Ketalar atropine sulfate and pentobarbital sodium. Animal models were divided into permanent cerebral ischemia models and cerebral ischemia-reperfusion models. Cerebral ischemia-reperfusion models, except 1 item [32], were made by krypton laser, and the rest were made by nylon thread embolization method. Most of the cerebral ischemia models were made by using laser-induced photochemical reactions in the middle cerebral artery ischemia model [15, 17, 18, 20, 22], and among the remaining, one was made by using the wire embolization method and two were made by using the cautery method. Seven studies observed pericyte morphology or capillary changes, 9 studies observed PDGFR *β*+ pericytes, 1 study observed both pericyte morphology and PDGFR *β*+ pericytes, and 4 studies observed *α*-SMA labeling positive cells (i.e., pericytes), and 1 study looked at VEGFR-3 expression. The duration of cerebral ischemia or reperfusion was 0.5 h, 1 h, 1.5, 3 h, 6 h, 12 h, 24 h, 48 h, 3 d, 4 d, 5 d, 7 d, 14 d, 28 d, and 30 d. The overall characteristics of the listed publications are shown in Tables 2–4.

### 3.4. Changes in Pericytes within 24 Hours after Ischemia/Reperfusion

Pericytes were separated from the basement membrane after 1 h of ischemia [18], and the number of *α*-SMA-labeled positive cells (pericytes) in the ischemic cortical area increased significantly with irregular morphology [34]. Swollen pericytes can be found at 1.5 h of ischemia [16]. The expression of pericyte marker *α*-SMA was reduced, and the expression of NG-2 was significantly reduced 24 h after ischemia [21].

After 2 h of ischemia and reperfusion for 6 h, the middle cerebral artery successfully reopened after 2 h of ischemia, and the pericytes remained constricted. *α*-SMA antibody-positive microvascular cells partially overlapped with NG-2 antibody positivity in the same cells [7]. At 24 h of cerebral ischemia-reperfusion, pericyte solute became swelled and oval-shaped, microvessels became compressed, and lumen became significantly smaller; pericytes were partially detached from the basement membrane [29].

### 3.5. Changes in Pericytes from 1 d-7 d after Ischemia/Reperfusion

PDGFR-*β* mRNA was upregulated obviously after 48 h of ischemia [17]. PDGFR *β*+ pericytes expressed on day 3 after stroke and started to increase on day 5 [19]. The expression of perivascular cell PDGFR-*β* expression in the infarct area was significantly upregulated within 5 days after MCAO [22].

After cerebral ischemia-reperfusion, PDGFR *β*+ pericytes were highly detached from brain endothelial cells and activated by the formation of several cellular protrusions entered the brain parenchyma through branch-like structures [35]. PDGFR *β*+ pericytes that were in the nonlethal ischemic injury zone began to express the NSC marker nestin at day 3 of ischemia. Some PDGFR *β*+ pericytes expressed the immature neuronal marker bicortin at day 7 [33]. The intensity of ipsilateral ischemic brain VEGFR-3 expression was significantly higher than before, and PDGFR *β*+ pericytes coverage was increased 3-7 days after focal cerebral ischemia [30]. PDGFR-*β* expression was highest on the fourth day after cerebral ischemia [32]. At 7 days of cerebral ischemia-reperfusion, in the ipsilateral hemisphere of MCAO injury, pericytes degenerated and compromised the integrity of the blood-brain barrier [25].

### 3.6. Changes in Pericytes after 7 d-30 d of Ischemia/Reperfusion

PDGFR *β*+ pericytes, which accumulated rapidly within 7-14 days, eventually occupied the entire infarcted area within 14-28 days [20]. No significant vascular-related expression of VEGF-3 was seen in the ischemic area 14 days after ischemia-reperfusion [26], and the expression of both PDGFR-*β* and CD31 was markedly reduced [31]. At 28 days after cerebral ischemia-reperfusion, *α*-SMA expression in the ischemic border zone was not obviously different from that in the sham-operated zone, and VEGFR-2 expression was increased [24]. At 30 days after cerebral ischemia-reperfusion, rats showed structural disruption of motor cortex microvessels and pericyte mutagenicity [23].

PDGFR-*β* expression was as below: low level at day 3, increased at day 7, and distributed in all infarct areas at day 14 [15]. After cerebral ischemia-reperfusion, pericyte coverage of endothelial cells in the ischemic brain region decreased transiently for transient ischemia, then increased, peaked at days 4-7, and then gradually returned to normal at day 28 [27].

## 4. Discussion

The results of this paper find that pericytes have different effects on cerebral microvasculature at different stages after stroke. Within 24 hours after ischemia, pericytes were damaged and dominated by vasoconstrictor function; from 2 to 7 days, pericytes showed a strong provascular regeneration function in the peri-ischemic region, which was the main stage of provascular regeneration; from 7 to 30 days, pericytes continued to perform provascular regeneration function at the range involving the whole ischemic region.

Within 24 hours after ischemia, pericytes are damaged, with predominantly constrictive vascular function. Constriction and death of pericytes are important pathogenic mechanisms in the early stages of cerebral ischemia. In the cerebral microvasculature, the regulation of blood supply is achieved by pericytes. Pericytes express large amounts of *α*-SMA contractile protein and have a contractile function similar to that of smooth muscle cells. The level of *α*-SMA in pericytes varies depending on their location. It is found that pericytes located at the ends of capillaries express the greatest amount of *α*-SMA [36], and the contractile capacity of these pericytes is particularly important in cerebral microarteries and capillaries that lack smooth muscle cells. Pericytes can also express tropomyosin, Desmin, and other contraction-related proteins [37, 38], which indicates that pericytes have a strong contractile capacity. Under physiological conditions, pericytes actively regulate cerebral blood flow through contraction and diastole. In pathological conditions, such as stroke, pericytes restrict blood flow by contraction and exacerbate ischemic injury. The contraction of pericytes observed in the ischemic stroke model suggests that the ischemic state can shift pericytes from diastolic to contractile. During stroke, pericyte constriction narrows the capillary lumen and prevents erythrocytes from passing normally through the constriction site, thereby impedes microcirculation [7]. Pericytes in vivo after ischemia constrict capillaries and die rapidly [39, 40]. The stiffness presented by pericytes death emerges a persistent capillary constriction that leads to a prolonged reduction in cerebral blood flow. Even arterial blood flow is restored, because of the excessive contraction of pericytes, the area of ischemia cannot restore blood flow [7]. It is found that in a rat model of focal cerebral ischemia, infarct volume could be reduced by preventing pericyte contraction [27]. Thus, modulating pericyte contraction and regulating microvascular blood flow early in ischemic stroke may be a good therapeutic entry point.

After 2 d of cerebral ischemia, expression of vascular growth-related factors in the ischemic region begins. After ischemic stroke, recanalization of occluded vessels is the fundamental solution [41, 42], while angiogenesis, which opens new blood flow pathways in hypoperfused areas, is essential for tissue protection and recovery [43]. Angiogenesis includes proliferation of vascular component cells, recruitment of pericytes, coverage of endothelial vessels by pericytes, and maturation of new vessels. The recruitment of pericytes is closely related to the barrier function of neovascularization. PDGF secreted by endothelial cells attracts pericytes and mediates the attachment and neonatal coverage of pericytes to form microvessels [44, 45]. Pericytes are easily susceptible to functional abnormalities and death in the cerebral ischemic environment [40], which leads to disruption of BBB integrity and consequently to ischemic brain tissue injury. Reduced pericyte coverage on capillaries can compromise vascular integrity. During recovery from ischemic brain injury, pericytes are recruited into newly formed immature vessels mainly through PDGF-*β* and PDGFR-*β* [46, 47]. In ischemic conditions, PDGFR-*β* is found to be specifically induced in peri-infarct pericytes. At the same time, peri-infarct endothelial cells can produce more PDGF-*β*. PDGF-BB/PDGFR-*β* signal is indispensable for the migration of pericytes to newly formed endothelial vessels and the formation of the BBB, which is an important step in microvascular maturation [22]. The recruitment of pericytes and subsequent coverage of endothelial cells reduces vascular leakage and stabilizes these vessels [1, 48]. The PDGF-BB/PDGFR-*β* pathway not only promotes pericyte proliferation, migration, survival, and attachment but also promotes the secretion of growth factors that affect angiogenesis. Pericytes control the balance and good secretion of endothelial PDGF-*β* and promote revascularization and maturation [49]. During ischemia and hypoxia, changes in PDGF-BB or PDGFR-*β* reduce the recruitment of pericytes [50], BBB destruction, and bleeding occurrence [51].

Compared with being given the critical role in angiogenesis [52], pericytes play a greater role in promoting stroke recovery. Important vascular growth factors, such as VEGF, have been identified in pericytes [53]. Both pericytes and endothelial cells produce VEGF which regulates angiogenesis in an autocrine and paracrine manner [54]. VEGF controls vascular growth during physiological and pathological processes and is an important regulator of protection and recovery from ischemic injury. After ischemic stroke, VEGF is expressed in the infarct zone; the expression of VEGF-A and its receptor VEGFR-2 is upregulated. VEGF-B is also involved in the angiogenic process after stroke by activating the VEGFR-1 signaling pathway [35]. The difference is that the action of VEGF-B does not affect the stability of the vasculature. Recombinant human vascular endothelial growth factor has been shown to increase capillary density and pericyte coverage, improve brain energy status and blood flow, and reduce infarct size in the MCAO model [55]. Pericytes play a beneficial role in ischemic stroke by promoting angiogenesis. Therefore, promote angiogenesis during recovery from cerebral ischemia and accelerate injury recovery, in which PDGF-*β* and VEGF can be important therapeutic targets.

The present study summarizes that pericytes are separated from the basement membrane after 1 hour of ischemia [18], and the number of *α*-SMA-labeled pericytes in the ischemic cortical area is significantly increased with irregular morphology [34]. Swollen pericytes can be found at 1.5 hours of ischemia [16]. Anti-*α*-SMA antibody-positive microvascular cells partially overlap with NG-2 antibody positivity in the same cells [7]. Pericyte marker *α*-SMA expression reduces, and NG-2 expression substantially reduces 24 h after ischemia [21]. At 28 days after cerebral ischemia-reperfusion, the expression of *α*-SMA in the ischemic border zone is not definitely different from that in the sham-operated zone [24]. After cerebral ischemia-reperfusion, pericyte coverage of endothelial cells in the ischemic brain region decreases briefly after transient ischemia, then increases, peaks at 4-7 days, and finally gradually recovers to normal at 28 days [27]. This indicates that *α*-SMA labeling of pericytes is elevated early in ischemia, and then, its expression gradually decreases after 24 hours of ischemia.

PDGFR *β*+ pericytes express at day 3 after stroke and start to increase at day 5 [19]. PDGFR-*β* expression in perivascular cells in the infarct area is significantly upregulated within 5 days after MCAO [22]. The intensity of ipsilateral ischemic brain VEGFR-3 expression is significantly high, and PDGFR *β*+ pericyte coverage increases 3-7 days after focal cerebral ischemia [30]. PDGFR-*β* expression was highest on the fourth day after cerebral ischemia [32]. The expression of PDGFR-*β* is at low level by day 3, increases by day 7, and distributes in all infarcted areas by day 14 [15]. At 28 days after cerebral ischemia-reperfusion, VEGFR-2 expression increases [24]. PDGFR-*β*, VEGFR-3, and VEGFR-2 expression start to increase on day 3, reach peak at days 3-7, and still express at day 18, indicates that pericytes have a predominantly provascular regenerative function in the middle and late stages of cerebral ischemia.

## 5. Conclusion

Pericytes exhibit different effects on cerebral microvasculature at different time points after stroke. In the early stage around 24 hours of ischemia-reperfusion, pericytes are damaged and constrict blood vessels to restrict blood flow and aggravate reperfusion injury, during which pericytes *α*-SMA and NG-2 can be selected as potential therapeutic targets. At the stage of cerebral ischemia for more than 3 days or recovery, pericytes promote vascular regeneration and accelerate the recovery of blood supply in the ischemic area, in which PDGFR-*β* and VEGFR can be used as potential therapeutic targets.

## Figures and Tables

**Table 1:** Methodological quality for each study included.

Study	A	B	C	D	E	F	G	H	I	J	Total
Makihara N et al. 2015 [15]	+	+	-	-	-	+	+	-	+	-	5
Liu KF et al. 2001 [16]	+	-	-	-	-	+	+	-	+	-	4
Renner O et al. 2003 [17]	+	-	-	-	-	+	+	-	+	-	4
Duz B et al. 2007 [18]	+	+	-	-	-	+	+	-	-	-	4
Sakuma R et al. 2016 [19]	+	-	-	-	-	+	+	-	-	-	3
Shibahara T et al. 2020 [20]	+	-	-	-	-	+	+	-	+	-	4
Zhang Y et al. 2020 [21]	+	+	-	-	-	+	+	-	+	+	6
Arimura K et al. 2012 [22]	+	+	-	-	-	+	+	-	+	+	6
Garbuzova-Davis S 2014 [23]	+	+	+	-	-	+	+	-	+	-	6
Zhai ZY et al. 2019 [24]	+	+	+	-	-	+	+	-	+	-	6
Garbuzova-Davis S et al. 2013 [25]	+	+	-	-	-	+	+	-	+	-	5
Shin YJ et al. 2013 [26]	+	+	-	-	-	-	+	-	+	-	4
Deguchi K et al. 2014 [27]	+	+	-	-	-	+	+	-	+	-	5
Noh JS et al. 2015 [28]	+	+	-	-	-	-	+	-	+	+	5
Han D et al. 2015 [29]	+	+	+	-	-	-	+	-	-	-	4
Zhou YF et al. 2018 [30]	+	+	-	-	-	+	+	-	+	+	6
Wang F et al. 2020 [31]	+	+	+	-	-	-	+	-	+	-	5
Nishimura A et al. 2016 [32]	+	+	+	-	-	+	+	-	+	+	7
Yemisci M et al. 2009 [7]	+	+	-	-	-	+	+	-	-	-	4
Nakata M et al. 2017 [33]	+	+	-	-	-	+	+	-	+	+	6
Tan QS et al. 2017 [34]	+	-	+	-	-	+	+	-	-	-	4
Jean LeBlanc N et al. 2018 [35]	+	+	-	-	-	+	+	-	+	-	5

Studies meeting the 10 criteria of risk of bias: A: peer-reviewed publications; B: temperature control; C: random assignment of treatments or controls; D: model-blind induction; E: blinded assessment of outcomes; F: use of anesthetics without significant intrinsic cardioprotective effects; G: appropriate animal models (elderly, diabetic, or hypertensive); H: sample size calculation; I: compliance with animal welfare regulations; J: potential conflict-of-interest statements.

**Table 2:** Changes in pericytes at different time points after cerebral ischemia.

Studies	Animals	Model	Ischemia time	Pericyte-related indicators
Makihara N et al. 2015 [15]	Male, wild-type S129 mouse, 20-35 g	MCAO (laser-induced photochemical reaction)	3 d, 7 d, 14 d, and 21 d	PDGFR-*β* expression: low levels before day 3; increased expression within day 7; after day 14 in all infarcted regions
Liu KF et al. 2001 [16]	Male, Sprague-Dawley rats, 300-350 g	MCAO (nylon suture)	1.5 h	Swollen pericytes
Renner O et al. 2003 [17]	OF1 mouse	MCAO (coagulated by bipolar diathermy)	3 h, 12 h, 48 h, 7 d, 12 d	PDGFR-*β* mRNA was specifically upregulated 48 h after ischemia.
Duz B et al. 2007 [18]	Male, Sprague-Dawley rats, 200-300 g	MCAO (cauterized with a microbipolar unit)	1 h, 3 h	Pericytes and basement membrane separated 1 h after ischemia
Sakuma R et al. 2016 [19]	Male, CB-17 mouse	MCAO (ligation)	3 d, 5 d, 7 d	PDGFR *β*+ pericytes express on day 3 poststroke and start to increase on day 5.
Shibahara T et al. 2020 [20]	Male, C57BL/6 mouse, 20-30 g	MCAO (laser-induced photochemical reaction)	1 d, 7 d, 14 d, 28 d	PDGFR *β*+ pericytes, which rapidly accumulate in 7-14 days and eventually occupy the entire infarcted area within 14 to 28 days
Zhang Y et al. 2020 [21]	Male, C57BL/6J mouse	MCAO (nylon suture)	24 h	The expression of the pericyte marker *α*-SMA was reduced, and the expression of NG-2 was significantly decreased.
Arimura K et al. 2012 [22]	Male, Wistar-Kyoto rats, 360-440 g	MCAO (laser-induced photochemical reaction)	1 d, 3 d, 5 d	The expression of PDGFR-*β* in perivascular cells in the peri-infarct area was significantly upregulated within 5 days after MCAO.

**Table 3:** Changes in pericytes at different time points of cerebral ischemia-reperfusion in rats.

Studies	Animals	Model	Ischemia time	Reperfusion time	Pericyte-related indicators
Garbuzova-Davis S et al. 2014 [23]	Male, SD rats, 260.5 ± 3.15 g	MCAO (filament)	60 min	30 d	Disruption of microvascular structure and degeneration of peripapillary cell protrusions in rat motor cortex
Zhai ZY et al. 2019 [24]	Male, SD rats, 280-320 g	MCAO (nylon suture)	90 min	28 d	The expression of *α*-SMA in the ischemic border zone was not significantly different from sham, and the expression of VEGFR-2 was increased.
Garbuzova-Davis S et al. 2013 [25]	Male, SD rats, 265.2 ± 1.49 g	MCAO (filament)	60 min	7 d	In the ipsilateral hemisphere of MCAO injury, pericytes degenerate and impair the integrity of the blood-brain barrier.
Shin YJ et al. 2013 [26]	Male, SD rats, 250-300 g	MCAO (nylon suture)	60 min	1 d, 3 d, 7 d, 14 d	The intensity of ipsilateral ischemic brain VEGFR-3 expression was significantly higher 3-7 days after focal cerebral ischemia. At 14 days after reperfusion, no significant expression of VEGFR-3 associated with blood vessels was seen in the ischemic area.
Deguchi K et al. 2014 [27]	Male, Wistar rats, 250-280 g.	MCAO (nylon suture)	90 min	1 d, 4 d, 14 d, 28 d	Coverage of endothelial cells by pericytes in ischemic brain areas decreases briefly and shortly after transient ischemia, then increases, peaks at 4-7 days, and then gradually returns to 28 days and six hours after reperfusion.
Noh JS et al. 2015 [28]	Male, SD rats, 250-300 g	MCAO (nylon suture)	60 min	6 h, 3 d, 7 d, 14 d	G-protein-coupled calcium-sensitive receptors are elevated in the ischemic region, preferentially affecting pericytes.
Han D et al. 2015 [29]	Male, Wistar rats, 250-280 g	MCAO (nylon suture)	120 min	24 h	The pericyte cytosol is swollen and oval in shape, compressing the microvasculature, and the lumen is significantly smaller; the pericyte is partially separated from the basement membrane.
Zhou YF et al. 2018 [30]	Male, SD rats, 300~600 g	MCAO (nylon suture)	2 h	1 d, 3 d, 7 d	PDGFR *β*+ pericyte coverage increased at 3 and 7 days after MCAO.
Wang F et al. 2020 [31]	Male, SD rats, 250–300 g	MCAO (nylon suture)	1.5 h	14 d	Pericapillary cell coverage plays a key role in maintaining blood-brain barrier integrity, and both PDGFR-*β* and CD31 expression were significantly reduced.

**Table 4:** Changes in pericytes at different time points of cerebral ischemia-reperfusion in mouse.

Studies	Animals	Model	Ischemia time	Reperfusion time	Pericyte-related indicators
Nishimura A et al. 2016 [32]	Male, WT(FVB/N) mouse, 20-35 g	MCAO (laser-induced)	60 min	1 d, 4 d, 7 d	PDGFR-*β* expression was highest on the fourth day after cerebral ischemia.
Yemisci M et al. 2009 [7]	Male, Swiss albino mouse	MCAO (nylon suture)	2 h	6 h	After 2 h of ischemia and reperfusion for 6 h, the middle cerebral artery successfully reopened after 2 h of ischemia, and the pericytes remained constricted. Anti-*α*-SMA antibody-positive microvascular cells partially overlapped with NG-2 antibody positivity in the same cells.
Nakata M et al. 2017 [33]	Male, CB-17 mouse	MCAO (nylon suture)	15 min, 20 min, 30 min	3 d, 5 d, 7 d	PDGFR *β*+ pericytes in the nonlethal ischemic injury zone begin to express the NSC marker nestin at day 3 of ischemia. Some PDGFR *β*+ pericytes express the immature neuronal marker bicortin at day 7.
Tan QS et al. 2017 [34]	Male, C57BL/6 mouse, 18-25 g	MCAO (nylon suture)	60 min	1 h	The number of *α*-SMA marker-positive cells (i.e., pericytes) in the ischemic cortical area was significantly increased, and the morphology was irregular.
Jean LeBlanc N et al. 2018 [35]	Male, C57BL6/j mouse	MCAO (nylon suture)	45 min	1 d, 4 d	PDGFR *β*+ pericytes are highly segregated from brain endothelial cells and activated by the formation of several cellular protrusions into the brain parenchyma through branching-like structures.

## Data Availability

The raw data used to support the findings of this study are available from the corresponding author upon request.
